# Pre-Anesthesia Extracorporeal Membrane Oxygenation in Two Lung Transplant Recipients with Severe Pulmonary Hypertension

**DOI:** 10.1155/2020/7265429

**Published:** 2020-02-12

**Authors:** Liu Minqiang, Gao Hong, Chen Jingyu, Wang Yanjuan, Xu Bo, Wang Guilong, Wu Qiang, Hu Chunxiao

**Affiliations:** ^1^Department of Anesthesiology, The Third People's Hospital of Shenzhen, No. 29 Bulan Road, Longgang District, Shenzhen, Guangdong 518112, China; ^2^Department of Anesthesiology, Wuxi People's Hospital, 299 Qingyang Road, Liangxi District, Wuxi, Jiangsu 214023, China; ^3^Department of Lung Transplantation Center, Wuxi People's Hospital, 299 Qingyang Road, Liangxi District, Wuxi, Jiangsu 214023, China

## Abstract

Extracorporeal membrane oxygenation (ECMO) is a widely used cardiopulmonary support method that is usually implemented after anesthesia during the period of lung transplantation (LTx). In severe pulmonary arterial hypertension (PAH) patients, however, anesthesia induction is a high-risk phase and can result in severe cardiorespiratory failure. Herein, we describe two severe PAH patients who received ECMO support before anesthesia and whose preoperative evaluations indicated that the risk was too high to safely survive the anesthesia induction period before LTx. The strategy was successful, and in both patients, hemodynamics was stable and no ECMO-related complications occurred.

## 1. Introduction

Severe pulmonary arterial hypertension (PAH) has been defined as mean pulmonary arterial pressure (mPAP) greater than 35 mmHg [1]. Patients with severe PAH are usually in a critical condition with right heart function on the verge of failure, and anesthesia management in these patients is challenging. During the induction period of anesthesia, the associated sudden sympathetic nerve depression and change in respiratory pattern can aggravate right heart dysfunction and cause severe chest tightness, dyspnea, and hypotension, and it can even lead to pulmonary hypertension crisis [2]. In an effort to avoid uncontrolled hemodynamic deterioration, the successful application of extracorporeal membrane oxygenation (ECMO) before general anesthesia induction has previously been reported [3]. To improve the safety of these patients during perioperative period, we decided to perform ECMO assistance before anesthesia induction in severe PAH patients whose mPAP was greater than 50 mmHg, or pulmonary artery systolic pressure (PASP) greater than 100 mmHg, and cardiac index (CI) less than 2 L/min/m [2]. We hope that this strategy will help some patients to survive the anesthesia induction period stably.

## 2. Case Presentation

The strategy described below was approved by the Medical Ethics Committee of Wuxi People's Hospital, Nanjing Medical University, Wuxi, Jiangsu, China (approval number KYLLH2018022). Written informed consent was obtained from each patient before anesthesia. Both patients were in a state of cardiorespiratory decompensation despite maximum pharmacological therapy.

### 2.1. Case 1

**Figure 1:**
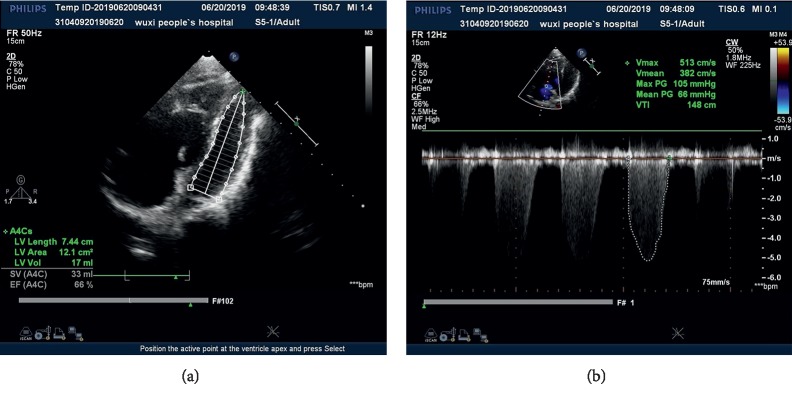
(a) Transthoracic echocardiography in patient 1 depicted substantial enlargement of the right atrium and right ventricle, and widening of the pulmonary artery. (b) Pulmonary artery systolic pressure increased to up to 120 mmHg.

A 22-year-old male was diagnosed as idiopathic pulmonary hypertension. Preoperative right cardiac catheterization revealed mPAP increased to up to 81 mmHg, CI dropped down to 1.98 L/min/m^2^ (Figures 1(a) and 1(b)), and B-type natriuretic peptide (BNP) rose to 1134 pg/ml.

### 2.2. Case 2

**Figure 2:**
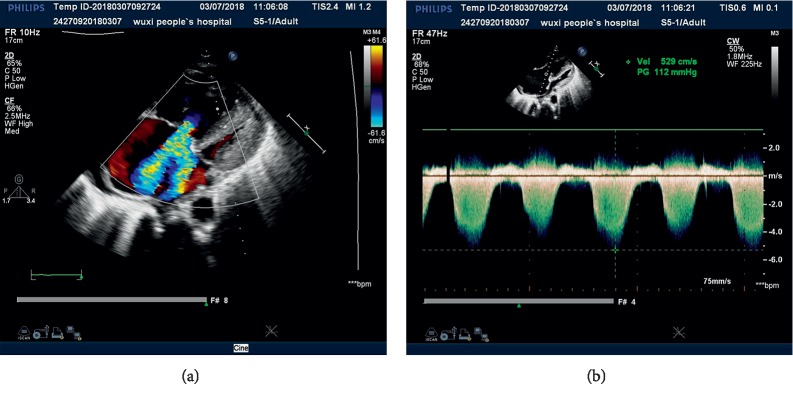
(a) Transthoracic echocardiography in patient 2 depicted obvious widening of the right atrium and right ventricle and severe regurgitation of the tricuspid valve. The pulmonary artery was distinctly broadened. (b) Pulmonary artery systolic pressure increased to up to 118 mmHg.

A 59-year-old female diagnosed with diopathic interstitial pneumonia. Preoperative right cardiac catheterization revealed mPAP rose up to 67 mmHg, CI fallen to 1.82 L/min/m^2^ (Figures 2(a) and 2(b)), and BNP ascended to 5328 pg/ml.

After patient admission, the intravenous medication of PAH was maintained via a catheterization of the right internal jugular vein obtained in the intensive care unit. Patients were kept awake, a half sitting position was utilized and peripheral arterial access was secured via a 22-G arterial puncture needle on the right radial hand. After local anesthesia took effect, a dose of heparin 50 U/kg was injected, and a venoarterial extracorporeal membrane oxygenator was implanted via a catheter from the right femoral artery up to the descending aorta for perfusion, and a second cannula from the right femoral vein to below the junction between the inferior vena cava and the right atrium for drainage, which were monitored with transesophageal echocardiography. During the period of ECMO bypass, the flow rate was adjusted within 1.5–3.0 L/min based on the patient's hemodynamic and oxygenation status, and the activated clotting time was monitored every 2 h, and maintained for 160–200 s by the administration of heparin 10–20 U/kg when necessary. In both patients, cardiopulmonary function largely stabilized within approximately 5 to 10 min with a small dose of infused norepinephrine; the preoperative therapy of PAH was removed, and general anesthesia induction began with an injection of midazolam, etomidate, sufentanil, and *cis*-atracurium. A Swan-Ganz catheter was then inserted via the right internal jugular vein to continuously monitor central venous pressure, mPAP, CI, systemic vascular resistance, and pulmonary vascular resistance. Bedside electrocardiography monitoring indicated that mPAP decreased by approximately 20–25 mmHg and CI rose to >2 L/min/m [2]. During the surgery, both patients remained hemodynamically stable, and arterial saturation was sufficient. In Case 1, the bilateral LTx completed successfully, mPAP was maintained at approximately 35 mmHg, and ECMO support was continued for 24 h after the surgery. On the other hand, ECMO was discontinued at the end of the surgery in Case 2 because only an unilateral LTx (the right side) was carried out and mPAP rapidly dropped down to 25 mmHg around. Both patients recovered without ECMO-associated complications, and neither exhibited primary graft dysfunction. In both patients, pulmonary artery systolic pressure decreased to 35–39 mmHg, and CI and BNP were at normal levels 3 months after the surgery. Cardiopulmonary function had recovered well in both patients at a 6-month follow-up time-point.

## 3. Discussion

The perioperative application of ECMO can effectively alleviate PAH, reduce right ventricular afterload, and treat right heart failure; thus, this supplementary therapy is often needed in severe PAH patients during LTx [4]. It has been reported that patients with severe PAH associated with lung disease usually had serious right ventricular insufficiency, and that survival was very poor [5]. Moreover, with the inhibitory effects of anesthetics on myocardium and the change in ventilation mode to positive pressure ventilation, pulmonary vascular resistance and ejection resistance of the right heart increase sharply in patients with severe PAH, and right heart dysfunction can occur rapidly, followed by pulmonary flow decline, left ventricular preload and output decrease, systemic hypotension, and even cardiac arrest [2,6]. The above-mentioned studies indicate that the induction period of general anesthesia is potentially fatal in patients with severe PAH, and it is very challenging for anesthesiologists to help these patients to undergo anesthesia induction smoothly.

We have described two critical patients with severe PAH, both of whom were with right cardiac failure primary or secondary to respiratory failure and had exhibited rapid deterioration of their disease requiring urgent LTx. Pre-anesthesia assessment suggested that their cardiopulmonary condition was too bad to tolerate direct anesthesia. However, it was noted that it had been reported that ECMO can occasionally be established with local anesthesia and light sedation before general anesthesia induction, to prevent hemodynamic instability [7]. To avoid further deterioration resulting in cardiorespiratory arrest, we managed ECMO bypass while the patients were still awake. With the assistance of ECMO, pulmonary vascular resistance decreased in both patients, but CI increased markedly, and this contributed to good conditions for the smooth implementation of anesthesia and the subsequent surgery.

In summary, the above-described procedure represents an unconventional strategy involving the provision of ECMO support before anesthesia induction in patients with severe PAH to avoid hemodynamic deterioration. More cases, case series, or comparative studies are needed to evaluate the outcomes associated with this approach, and a well-constructed evaluation scale such as one incorporating the limits of mPAP and CI should also be adopted to assess postoperative complications.
